# hPER3 promotes adipogenesis via hHSP90AA1-mediated inhibition of Notch1 pathway

**DOI:** 10.1038/s41419-021-03584-0

**Published:** 2021-03-19

**Authors:** Xinxing Wan, Liyong Zhu, Liling Zhao, Lin Peng, Jing Xiong, Wenjun Yang, Jingjing Yuan, Fang Liang, Keke Zhang, Ke Chen

**Affiliations:** 1 Department of Endocrinology, Third Xiangya Hospital, Central South University, Changsha, Hunan 410013 China; 2 Department of Bariatric and Metabolic Surgery, Third Xiangya Hospital, Central South University, Changsha, Hunan 410013 China; 3 Department of Nephrology, The First Hospital of Changsha, Changsha, Hunan 410005 China

**Keywords:** Molecular biology, Obesity

## Abstract

The period circadian regulator 3 (PER3) has been reported to play a negative role in human immortalized bone marrow-derived Scp-1 cells (iBMSCs) and patient adipose-derived stromal cells (PASCs) or a negative/positive role in mice adipogenesis. However, human PER3 (hPER3) was identified as a positive regulator of human adipose tissue-derived stromal cells (hADSCs) adipogenesis in this study. Silencing or overexpression of hPER3 in hADSCs inhibited and promoted adipogenesis in vitro. In vivo, the overexpression of hPER3 increased high-fat diet-induced inguinal white adipose tissue (iWAT) and epididymal white adipose tissue (eWAT) forms, increasing systemic glucose intolerance and insulin resistance. Molecularly, hPER3 does not interact with hPPARγ, but represses Notch1 signaling pathway to enhance adipogenesis by interacting with hHSP90AA1, which is able to combine with the promoter of hNotch1 and inactivate its expression. Thus, our study revealed hPER3 as a critical positive regulator of hADSCs adipogenesis, which was different from the other types of cells, providing a critical role of it in treating obesity.

## Introduction

The participation of circadian rhythm in metabolic regulation has been extensively studied and circadian rhythm gene dysfunction induces metabolic disorders such as obesity, insulin resistance, diabetes, etc^1–5^. At molecular level, the genes of circadian rhythm form a transcriptional and posttranscriptional feedback loop. Briefly, the circadian locomotor output cycles kaput gene (*CLOCK*) and the aryl hydrocarbon receptor nuclear translocator like (ARNTL), which are often called as the brain and muscle Arnt like protein-1 (BMAL1) forms, promote the expression of period circadian regulators (PERs, including PER1, PER2, and PER3) and cryptochrome (CRYs, including CRY1 and CRY2). These in turn enhance the expression of downstream genes and, at the same time, the PERs and CYRs also form a heterodimer and then translocate into the nucleus to negatively inhibit the expression of CLOCK/ARNTL^6^.

PER1, PER2, and PER3 are three important components of the PERs family. Although they have similar structure, they play different role in adipogenesis. Also there are no studies that discussed the participation of PER1 in adipogenesis, but previous studies have shown that PER2 played a contradictory role in adipogenesis. Boucher et al.^7^ have proved that human PER2 (hPER2) positively regulated adipogenesis in human primary bone marrow mesenchymal stems cells (hBMSCs), but the mouse PER2 (mPER2) negatively regulated adipogenesis in mouse embryo fibroblasts (MEFs) and 3T3-L1 preadipocytes^8^. With regard to PER3, inconsistent results have been reported on its role in the regulation of adipogenesis, although three studies have shown that mPER3 negatively regulates adipogenesis^9–11^ and two studies also proved hPER3 repressed adipogenesis in human MSCs^9,12^. Gradual downregulation of hPER3 was found during the adipogenesis of human immortalized bone marrow-derived Scp-1 cells (iBMSCs) and patient adipose-derived stromal cells (PASCs)^12^, but a recent study showed that knockout of mPER3 decreased the body weight gain after high-fat diet, showing a positive regulatory behavior^13^. The molecular mechanism of mPER3 showed inhibition of peroxisome proliferator-activated receptor γ (PPARγ), which is a critical transcription factor of adipogenesis, via interacting with PPARγ response element, and also mPER3 repressed the Kruppel-like factor 15, which is a crucial positive regulator of adipogenesis^9,11,14^. Hitherto, the molecular mechanism as to how hPER3 regulates adipogenesis still has not been reported.

Notch1 signaling is an evolutionarily conserved intercellular signaling pathway, which extensively participates in differentiation and development of cells, tissues, and organs by regulating adjacent cells, which in turn regulates metabolism, cancer, immunity, and so on^15–18^. Some studies have also shown that hPER3 negatively regulates Notch1 signaling pathway, e.g., enhancement of hPER3 has markedly inhibited Notch1 and JAG1 expression in human colorectal cancer stem-like cells and prostate cancer cells^19,20^. However, Notch pathway plays a significantly different role in adipogenesis. Although most of the studies showed that Notch signaling pathway negatively regulates adipogenesis^21–25^, some studies have shown that Notch pathway positively regulates adipogenesis^26,27^ or it is not essential for adipogenesis^28^, and these contradictory results were reviewed by Shan et al^29^. In addition, repression of Notch pathway promoted PPARγ expression^25,27,30–32^, but the underlying molecular mechanism still remained unclear.

We herein showed that hPER3 positively regulated human adipose tissue-derived stromal cells (hADSCs) adipogenesis via repressing human Notch1 (hNotch1) signaling pathway. Moreover, hPER3 during hADSCs adipogenesis was gradually upregulated and could not interact with hPPARγ protein. hPER3 represses hNotch1 expression by combining with hHSP90AA1, which can interact with hNotch1 promoter and thereby suppress hNotch1 expression. Our study demonstrated hPER3 as a positive regulator in adipogenesis and it is beneficial for better understanding of the molecular mechanism of circadian rhythm genes that participate in adipocyte development.

## Results

### hPER3 was upregulated in adipose tissues of obese individuals and mice and during adipogenesis

**Fig. 1:**
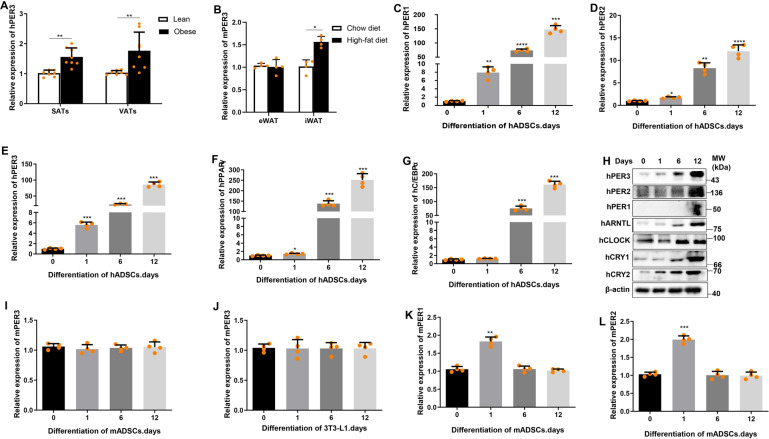
hPER3 was upregulated during adipogenesis and adipose tissue in obese individuals. **A** hPER3 mRNA expression of SATs and VATs in lean and obese individuals, *n* = 8 biologically independent samples. **B** mPER3 mRNA expression in eWAT and iWAT after C57BL/6 mice were fed with Chow or high-fat diet for 12 weeks (Chow diet, *n* = 4, high-fat diet, *n* = 4). **C**–**G** hPER1, hPER2, hPER3, hPPARγ, and hCEBP/α mRNA expression during induction of hADSCs to mature adipocytes for 12 days (*n* = 4). **H** The protein expression of circadian rhythm system including hPER1, hPER2, hPER3, hARNTL, hCLOCK, hCRY1, and hCRY2 during hADSCs adipogenesis for 12 days (*n* = 3). **I**, **J** mPER3 mRNA expression during mADSCs and 3T3-L1 cells were induced to mature adipocytes for 12 days (*n* = 4). **K**, **L** mPER1 and mPER2 mRNA expression during mADSCs was induced to mature adipocytes for 12 days (*n* = 4). Error bars are presented as means ± SD. Student’s *t*-test for **A** and **B**, one-way ANOVA with Dunnett’s post hoc test for **C**–**G** and **I**–**L**. Statistical significance was presented as *p < 0.05, **p < 0.01, ***P < 0.001, and ****P < 0.0001.

To first explore the expression of hPER3 in abdominal subcutaneous adipose tissues (SATs) and visceral adipose tissues (VATs) of lean and obese individuals, the hPER3 mRNA levels were detected using quantitative reverse transcriptase PCR (qRT-PCR). The results revealed that both SATs and VATs were highly expressed in obese (body mass index (BMI) ≥ 32) individuals when compared to lean individuals (BMI < 18.5) (Fig. 1A). Next, the mPER3 mRNA of 24 h circadian rhythm level in C57BL/6 inguinal white adipose tissue (iWAT) was detected, mPER3 mRNA expression was oscillated with time change (Supplementary Fig. S1A), and we collected mice samples at 8 h of circadian time for all mice experiments. The mPER3 mRNA expression in epididymal WAT (eWAT) and iWAT was detected after the C57BL/6 mice were fed with high-fat diet for 12 weeks, and our results revealed that the mPER3 expression showed no change in eWAT, but was highly expressed in iWAT (Fig. 1B). The hADSCs were then isolated from abdominal SATs of 4 normal BMI (18.5–23.9) patients and mature adipocytes were induced. The mRNA expression of hPER1, hPER2, hPER3, hCLOCK, hARNTL, hCRY1, and hCRY2, and markers of mature adipocytes hPPARγ and hCEBP/α, were then detected. Surprisingly, these genes were shown to be upregulated during adipogenesis (Fig. 1C–G and Supplementary Fig. S1B–E). In addition, the protein expression level of circadian rhythm pathway was verified and hPER1, hPER2, hPER3, hCLOCK, hARNTL, hCRY1, and hCRY2 showed a gradual upregulation during adipogenesis (Fig. 1H). These results implied that the hPER3 might positively regulate adipogenesis, but the study conducted by Knarr et al.^12^ showed that the expression of hPER3 was gradually downregulated during adipogenesis of human iBMSCs and PASC, validating hPER3 negatively regulated adipogenesis. In addition, three studies showed negative mPER3 and a study showed positive regulation of mPER3 in adipogenesis in a mice model^9–11,13^. Due to contradictory results, the expression of mPER3 during mice ADSCs (mADSCs) and 3T3-L1 adipogenesis in mRNA level was finally explored, and the results showed that mPER3 did not change in mADSCs and 3T3-L1 (Fig. 1I, J), but mPER1 and mPER2 were significantly upregulated in the early stage (day 1), and showed no change in the middle and later stages (day 6 and day 12) (Fig. 1K, L). Overall, our results revealed some differences from the results of the previous studies, implying that hPER3 plays a different role from mPER3 in adipogenesis.

### hPER3 positively regulates adipogenesis

**Fig. 2:**
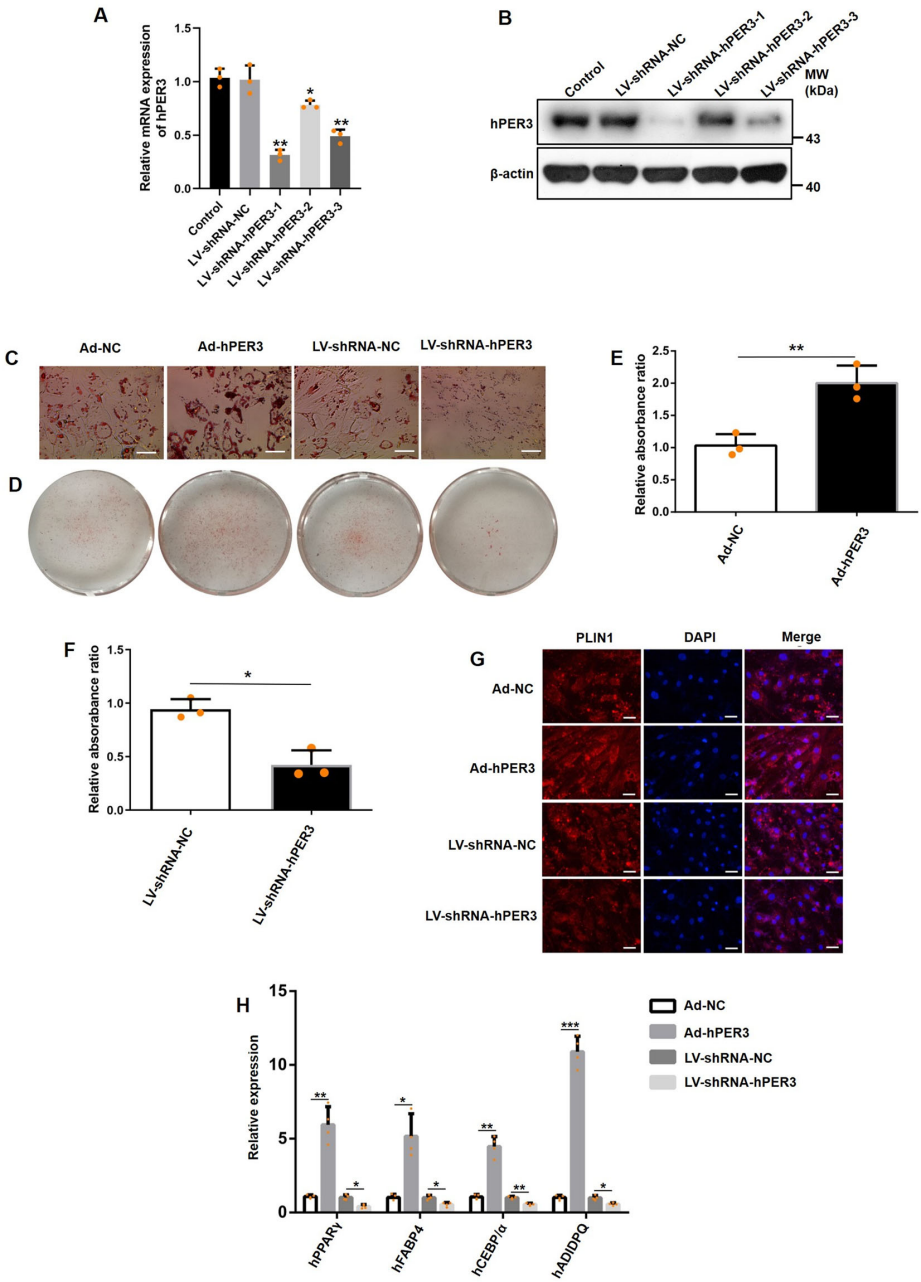
hPER3 is a positive regulator of adipogenesis. **A**, **B** The translation efficiency of LV-shRNA-hPER3 was evaluated by RT-qPCR (**A**) and western blotting (**B**). **C** The cellular lipid droplets were assessed using Oil Red O staining. The hADSCs were transfected with Ad-NC, Ad-hPER3, LV-shRNA-NC, and LV-shRNA-hPER3, induced for 12 days, and then assessed by Oil Red O staining. Scale bar = 100 μm. *n* = 3 per group. **D** Experimental settings are similar to **C** and images were captured by a camera, *n* = 3 per group. **E**, **F** The relative absorbance ratio. The cellular lipid droplets content was obtained by absorbance quantification at 520 nM, *n* = 3. **G** Immunofluorescence analysis of PLIN1. The hADSCs were transfected with Ad-NC, Ad-hPER3, LV-shRNA-NC, and LV-shRNA-hPER3, induced for 12 days, and PLIN1 was assessed using immunofluorescence analysis. Scale bar = 100 μm. *n* = 3 per group. **H** The expression of mature adipogenic genes in hADSCs of each group that were induced for 12 days, respectively. *n* = 4. Data are presented as means ± SD. Student’s *t*-test, *p < 0.05, **p < 0.01, and ***P < 0.001.

To further explore whether hPER3 negatively or positively regulates adipogenesis, adenoviral hPER3 (Ad-hPER3) and lentiviral shRNA-hPER3 (LV-shRNA-hPER3) were transfected into hADSCs for 72 h, respectively. The translation efficiency of LV-shRNA-hPER3 was evaluated by RT-qPCR and western blotting (Fig. 2A, B). hADSCs were subsequently induced into mature adipocytes. The lipid droplets were observed through Oil Red O staining and were calculated with relative absorbance ratio. The results showed that overexpression of hPER3 has strikingly increased lipid accumulation and, in contrast, only small lipid droplets were formed in the hPER3 knockdown group (Fig. 2C, D). Similar results were verified in relative absorbance ratio (Fig. 2E, F). Next, the lipid droplets were also evaluated with perilipin 1 (PLIN1) expression using immunofluorescence (Fig. 2G). Finally, some markers in mature adipocytes including PPARγ and CEBP/α, fatty acid binding protein 4 (FABP4), and adiponectin (ADIPOQ) were detected, and these genes were significantly upregulated when hPER3 was overexpressed after 12 days of hADSCs induction. In contrast, these were drastically downregulated in the LV-shRNA-hPER3 group (Fig. 2H). These results suggested that hPER3 positively regulates adipogenesis.

### hPER3 regulates the expression of hPPARγ

**Fig. 3:**
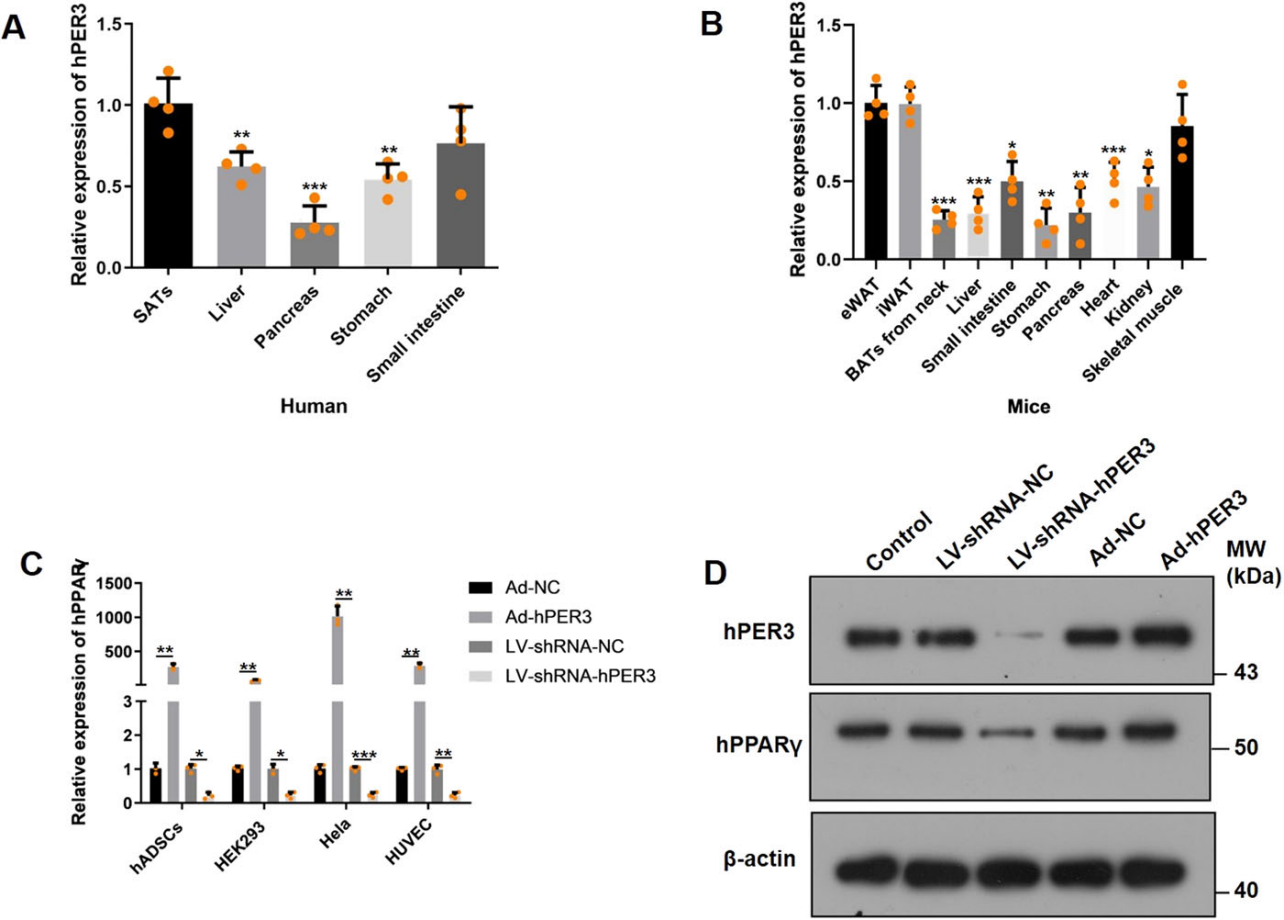
Expression of hPER3 and mPER3 in different tissues and hPER3 promotes hPPARγ. **A** The expression of hPER3 in human tissues, *n* = 4, and the relative expression was compared to SATs. **B** The expression of mPER3 in mice tissues, *n* = 4, and the relative expression was compared to eWAT. **C** The mRNA expression of hPPARγ after hADSCs, HEK293, HeLa, and HUVECs were transfected with Ad-NC, Ad-hPER3, LV-shRNA-NC, and LV-shRNA-hPER3, respectively, *n* = 3. **D** The protein level of hPPARγ expression was evaluated by western blotting in control (untransfected hADSCs) and hADSCs transfected with Ad-NC, Ad-hPER3, LV-shRNA-NC, and LV-shRNA-hPER3 for 72 h, *n* = 3. Data are presented as means ± SD. Student’s *t*-test, *p < 0.05, **p < 0.01, and ***P < 0.001.

To further explore the molecular mechanism of hPER3 regulation of adipogenesis, the mRNA expression of PER3 in different tissues of humans and mice was detected. In humans, the mRNA expression of hPER3 was higher than that of other tissues, including the liver, pancreas, stomach, and small intestine, in four individual patients (Fig. 3A) and similar results also showed that the mPER3 expression of eWAT, iWAT, and skeletal muscle was higher than that of the brown adipose tissues, liver, pancreas, stomach, small intestine, heart, and kidney in mouse (Fig. 3B). A previous study has verified that mPER3 interacts with mPPARγ and inhibits its expression^9^, and whether overexpression or silencing of hPER3 can regulate hPPARγ was also explored in four types of human cells—hADSCs, HEK293, Hela, and HUEVC. Surprisingly, overexpression of hPER3 was shown to remarkably upregulate hPPARγ in mRNA level, but silencing of hPER3 strongly repressed hPPARγ (Fig. 3C). Moreover, the hPPARγ protein-level change was same as that of the mRNA level detected in hADSCs (Fig. 3D) and these results elucidated that hPER3 positively regulated the expression of hPPARγ, and even hADSCs did not induce to mature adipocytes. In addition, hPER3 did not affect hSREBF1, which is another transcription factor of lipogenesis, and hCEBP/α at mRNA levels (Supplementary Fig. S2A, B). Next, to elucidate the molecular mechanism as to why hPER3 and mPER3 play different roles in regulating the adipogenesis, some protein sequences of circadian rhythm pathway in different animals were first compared, and only hPER2 and hPER3 showed significant differences with other animals. For instance, *Mus musculus* and *Rattus norvegicus* (Supplementary Table 1). However, hPER2 and hPER3 are highly conserved in some primate animals, e.g., *Pan troglodytes*, *Hylobates moloch*, *Pongo abelii*, and *Nomascus leucogenys* (Supplementary Table 1). hPER1, hCLOCK, and hARNTL are very highly conserved and similar protein sequences were observed in both primates and other animals (Supplementary Table 1). Our results showed significant differences in protein sequences of hPER2 and hPER3 when compared to other animals, which might lead to different biological functions.

### hPER3 negatively regulates Notch1 pathway

**Fig. 4:**
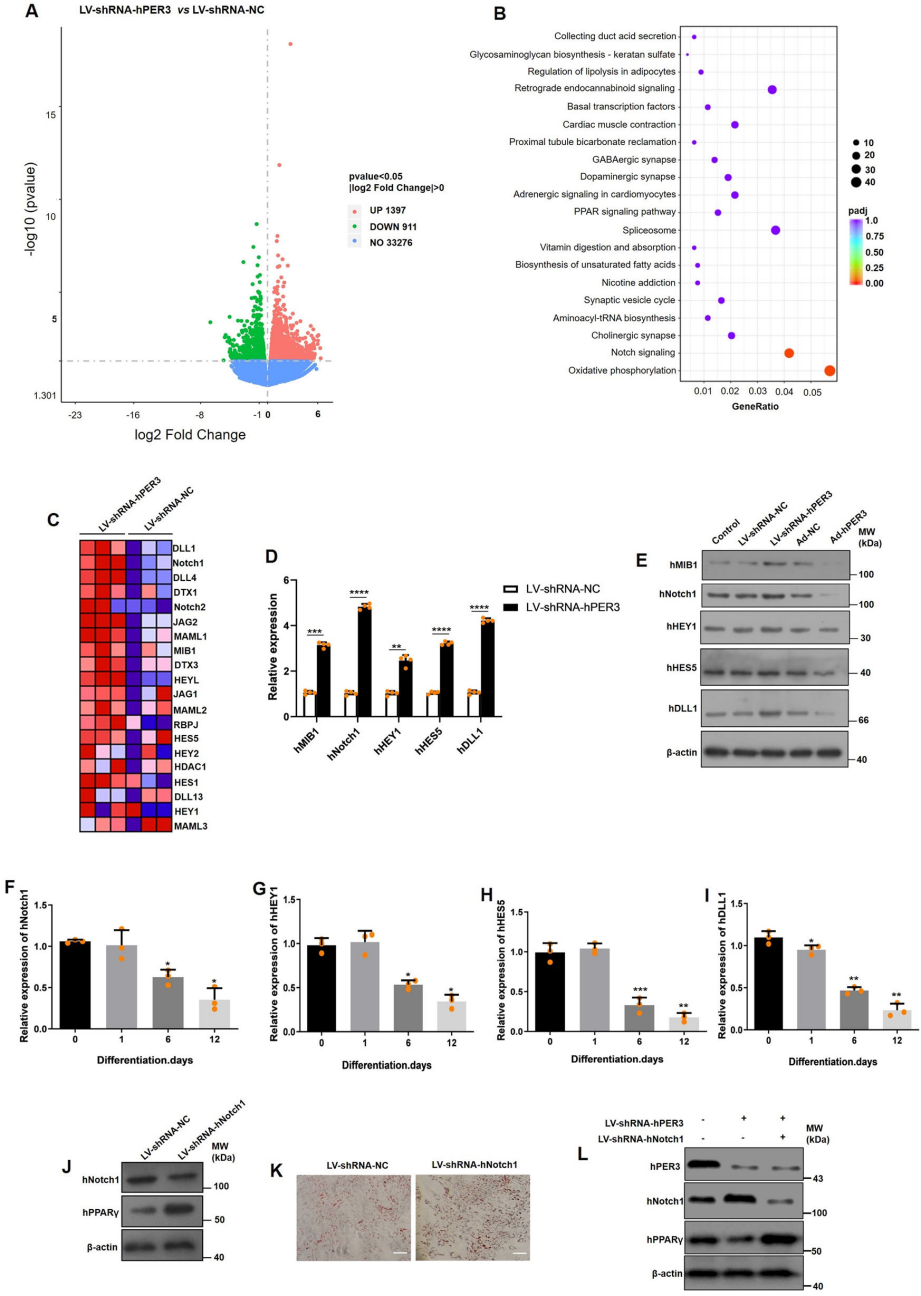
hPER3 repressed Notch1 pathway. **A** The volcano plot of RNA-seq showing differential gene expression after hADSCs were transfected with LV-shRNA-NC and LV-shRNA-hPER3, respectively. *n* = 3. **B** The pathway analysis of RNA-seq after hADSCs were transfected with LV-shRNA-NC and LV-shRNA-hPER3, respectively. The point size represents the enriched gene numbers, red points represent the upregulation pathway, and blue points represent the downregulated pathway. **C** Heatmap of Notch pathway representing the relative genes expression. Red and blue represent upregulation and downregulation expression, respectively. **D** The mRNA expression of Notch1 pathway after hADSCs were transfected with LV-shRNA-NC and LV-shRNA-hPER3, *n* = 4. **E** The protein-level expression of Notch1 pathway was evaluated in Control (untransfected hADSCs) and hADSCs were transfected with Ad-NC, Ad-hPER3, LV-shRNA-NC, and LV-shRNA-hPER3 for 72 h, *n* = 3. **F**–**I** The hNotch1, hHEY1, hHES5, and DLL1 mRNA expression during hADSCs adipogenesis, *n* = 3. **J** Silencing of hNotch1 promoted the expression of hPPARγ at the protein level, *n* = 3. **K** The lipid droplets were assessed with Oil Red O staining. hADSCs was transfected by LV-shRNA-NC and LV-shRNA-hPER3, and then the cells were induced for 7 days, *n* = 3. Scale bar = 200 μm. **L** After simultaneous transfection with LV-shRNA-hPER3 and LV-shRNA-hNotch1 in hADSCs for 72 h, hNotch1 and hPPARγ levels were detected, *n* = 3. Data are presented as means ± SD. Student’s *t*-test for **D**, one-way ANOVA with Dunnett’s post hoc test for **F**–**I**. *p < 0.05, **p < 0.01, and ***P < 0.001.

To explore hPER3 regulation in adipogenesis, an RNA sequencing (RNA-seq) array was used to investigate differential gene expression after hADSCs were transfected by lentiviral shRNA-NC (LV-shRNA-NC) and LV-shRNA-hPER3 for 72 h. Total RNA was collected and the results of RNA-seq showed that 1397 genes were upregulated and 911 genes were downregulated in LV-shRNA-hPER3 when compared to LV-shRNA-NC (Fig. 4A and Supplementary Fig. S3A). Gene Ontology (GO) analysis showed that the monovalent inorganic cation transmembrane, dendritic tree, axon, etc., were significantly enriched (Supplementary Fig. S3B) in pathway analysis, especially the Notch and oxidative phosphorylation pathways showed significant enrichment and upregulation (Fig. 4B). In addition, some pathways that participated in regulating lipolysis, adipogenesis, and biosynthesis of unsaturated fatty acids were enriched and downregulated (Fig. 4B). In Notch pathway, 20 genes, such as *DLL1*, *Notch1*, *Notch2*, and *HES5*, were upregulated (Fig. 4C). The differential gene expression of Notch signaling pathway in RNA-seq was verified using qRT-PCR and found that hNotch1 mRNA was dramatically upregulated in LV-shRNA-hPER3 when compared to LV-shRNA-NC, and so Notch1 pathway was chosen for subsequent experiments (Fig. 4D). Next, the protein-level expression of Notch1 pathway was verified after overexpression of hPER3 or silenced in hADSCs, and these results indicated that the proteins of Notch1 pathway, for instance, hMIB1, hNotch1, hHEY1, hHES5, and hDLL1, were all downregulated after hPER3 was overexpressed and, on the contrary, these proteins were significantly upregulated after hPER3 silencing (Fig. 4E). Several studies have shown that Notch signaling pathway negatively regulates adipogenesis^21–24^ and downregulates PPARγ^27,30,32,33^, but few studies have reported reverse results^26,27^. Thus, the role of Notch1 signaling pathway in adipogenesis was verified in an hADSCs model and so the mRNA expression of hNotch1, hHEY1, hHES5, and hDLL1 during adipogenesis was first detected. These genes were gradually downregulated, which indicated negative regulation of Notch1 pathway in hADSCs adipogenesis (Fig. 4F–I). After transfection with lentiviral shRNA-hNotch1 (LV-shRNA-hNotch1) in hADSCs, the translation efficiency of LV-shRNA-hNotch1 was evaluated by RT-qPCR and western blotting (Supplementary Fig. 3C, D), hPPARγ levels were significantly promoted in hADSCs (Fig. 4J), and silencing of hNotch1 also increased adipogenesis via Oil Red O staining (Fig. 4K). Lastly, after simultaneous transfection with LV-shRNA-hPER3 and LV-shRNA-hNotch1 in hADSCs for 72 h, hNotch1 and hPPARγ levels were detected. This rescue experiment confirmed that silencing of hNotch1 was able to rescue the expression of hPPARγ (Fig. 4L). These results suggested that hPER3 can repress Notch1 pathway, subsequently upregulating hPPARγ and positively regulating adipogenesis.

### hPER3 regulates hNotch1 via interacting with hHSP90AA1

**Fig. 5:**
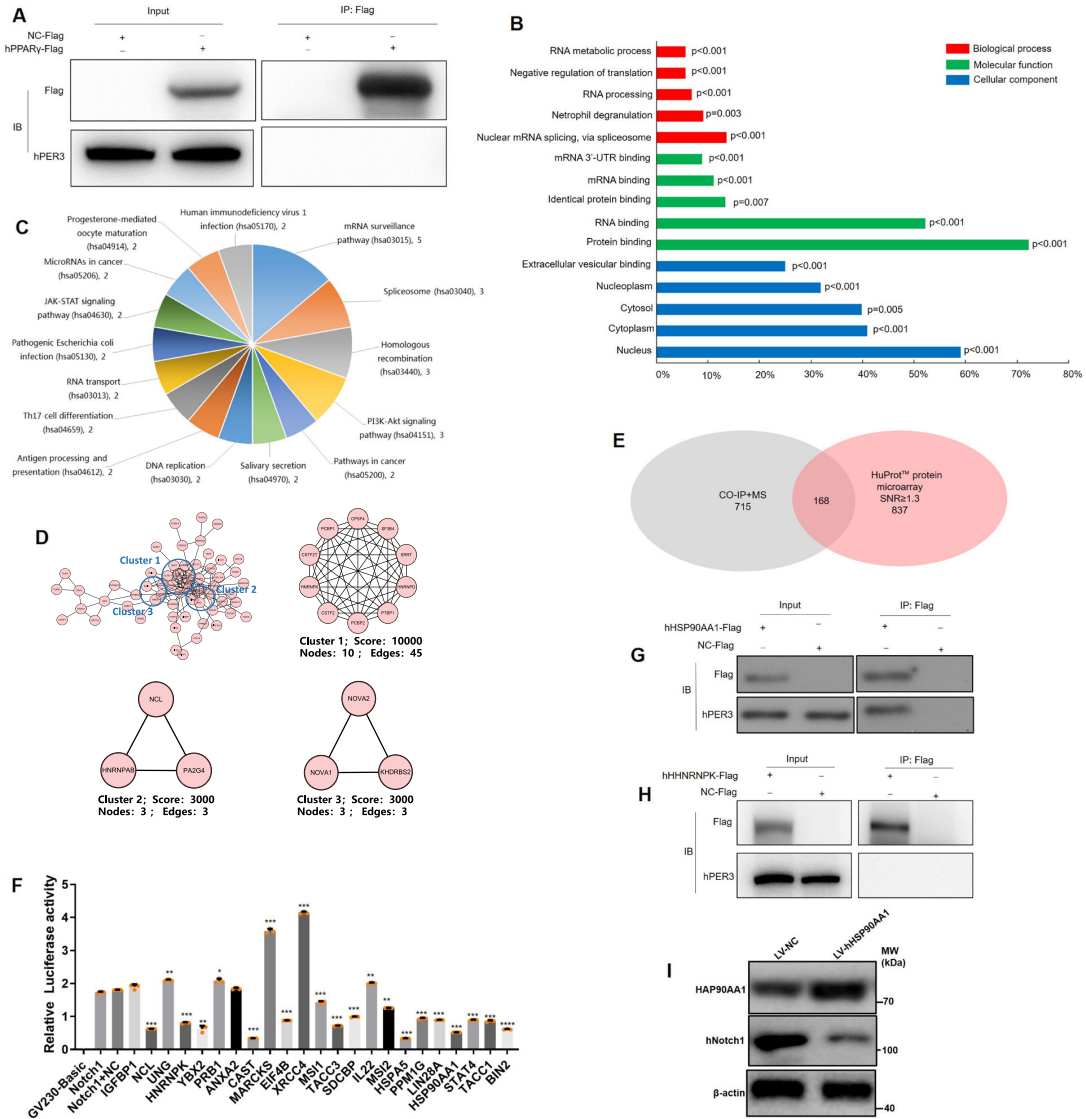
hPER3 interacts with hHSP90AA1 that combines with promoter of hNotch1. **A** hPER3 does not interact with hPPARγ using Co-IP, *n* = 3. **B** GO, **C** pathway, and **D** protein interaction and clustering analysis in HuProt™ protein microarray. **E** The overlap proteins in Co-IP + MS analysis and HuProt™ protein microarray. **F** The proteins directly combine with the promoter of hNotch1 via dual-luciferase reporter gene assay analysis, *n* = 3, and the relative expression was compared to the Notch1 group. **G** hPER3 interaction with hHSP90AA1 was evaluated using Co-IP, *n* = 3. **H** hPER3 does not interact with hHNRNPK using Co-IP, *n* = 3. **I** The hNotch1 expression was evaluated after transfection of LV-hHSP90AA1, *n* = 3. Data are presented as means ± SD. Student’s *t*-test, *p < 0.05, **p < 0.01, and ***P < 0.001.

To explore the mechanism of hPER3 regulation of Notch1 signaling pathway, we first verified whether hPER3 protein directly interacts with hPPARγ protein using co-immunoprecipitation (Co-IP) and this is because previous study has shown interaction of mPER3 with PPARγ^9^. However, our results showed that hPER3 and hPPARγ showed no interaction (Fig. 5A), indicating the difference in interaction of hPER3 from mPER3 with PPARγ. Second, to look for interaction proteins with hPER3, Co-IP + mass spectrometry (MS) analysis were conducted and a total of 715 possible interaction proteins were found, including some important regulatory genes on adipogenesis, e.g., *LMNB1*, *PARP1*, *FASN*, and *IL22*, etc. (Table 1). Third, about 2000 bp hNotch1 promoter plasmid was constructed and the combination proteins with hNotch1 promoter were detected with HuProt™ protein microarray. A total of 837 possible proteins of signal-to-noise ratio (SNR) ≥ 1.3 were chosen, for instance, IGFBP1, YBX2, ANXA2, MARCKS, and MSI1, etc., possibly combined with the promoter of hNotch1. GO analysis showed that these proteins regulate nuclear mRNA splicing via spliceosome, protein binding, and nuclear component, etc. (Fig. 5B). Pathway analysis showed that several genes particularly in regulation of mRNA surveillance and PI3K-Akt signaling pathway et al, (Fig. 5C), and the protein interaction and clustering analysis including via spliceosome (GO:0000398), nucleic acid binding (GO:0003676) and mRNA processing (GO:0006397) were summarized in Fig. 5D. Fourth, the overlap of 168 genes from two data sets of Co-IP + MS analysis and HuProt™ protein microarray were found (Fig. 5E). The above results provided us some potential genes that connect with hPER3 and hNotch1.

**Table 1:** Partial interaction of protein with hPER3 in Co-IP + MS.

Protein names	Gene names	*Q*-value	Score
Myosin-9	*MYH9*	0	323.31
Poly [ADP-ribose] polymerase 1	*PARP1*	0	323.31
Desmoplakin	*DSP*	0	323.31
Lamin-B1	*LMNB1*	0	323.31
Myosin-10	*MYH10*	0	323.31
Fatty acid synthase	*FASN*	0	323.31
protein kinase, DNA-activated, catalytic subunit	*PRKDC*	0	323.31
Pyruvate kinase	*PKM*	0	323.31
Vimentin	*VIM*	0	323.31
Tubulin β-class I	*TUBB*	0	307.24
Tubulin α-1B chain; Tubulin α-4A chain	*TUBA1B; TUBA4A*	0	284.42
Actin γ1	*ACTG1*	0	278.43
Heat shock protein HSP 90β	*HSP90AB1*	0	243.69
Clathrin heavy chain	*CLTC*	0	214.15
ATP-dependent RNA helicase A	*DHX9*	0	206.06
Heterogeneous nuclear ribonucleoprotein M	*HNRNPM*	0	203.83
Eukaryotic initiation factor 4A-I	*EIF4A1*	0	202.52
ATP synthase, H + transporting mitochondrial F1 complex, β-subunit	*ATP5B*	0	201.79
Cytoplasmic dynein 1 heavy chain 1	*DYNC1H1*	0	198.38

To further verify some proteins from 168 possible molecules whether they actually combine with the promoter of hNotch1, 23 proteins that are related with adipogenesis were chosen and shifted. Twenty-three recombinant plasmids were constructed and tested by dual-luciferase reporter gene assay, respectively. Our results showed that most of these proteins can combine with the promoter of hNotch1, including hHNRNPK, hHSP90AA1, MARCKS, XRCC4, etc. (Fig. 5F). hHNRNPK and hHSP90AA1 were finally chosen for a subsequent study. Next, to explore whether hHNRNPK or hHSP90AA1 interact with hPER3 protein, Co-IP experiment was performed. The results showed that hHSP90AA1 actually interacts with hPER3 protein (Fig. 5G) but hHNRNPK cannot combine with hPER3 (Fig. 5H). To explore whether hHSP90AA1 can regulate hNotch1, hADSCs were overexpressed with hHSP90AA1 and the hNotch1 protein level was significantly downregulated (Fig. 5I). However, hHSP90AA1 did not change during hADSCs adipogenesis (Supplementary Fig. S3E). In addition, we also found that hHSP90AA1 did not interact with hHNRNPK (Supplementary Fig. S4A). Our results suggested that hPER3 can interact with hHSP90AA1 and this hHSP90AA1 can combine to the promoter of hNotch1, thus acting as a bridge between hPER3 and hNotch1.

### hPER3 promotes iWAT and eWAT adipogenesis in vivo

**Fig. 6:**
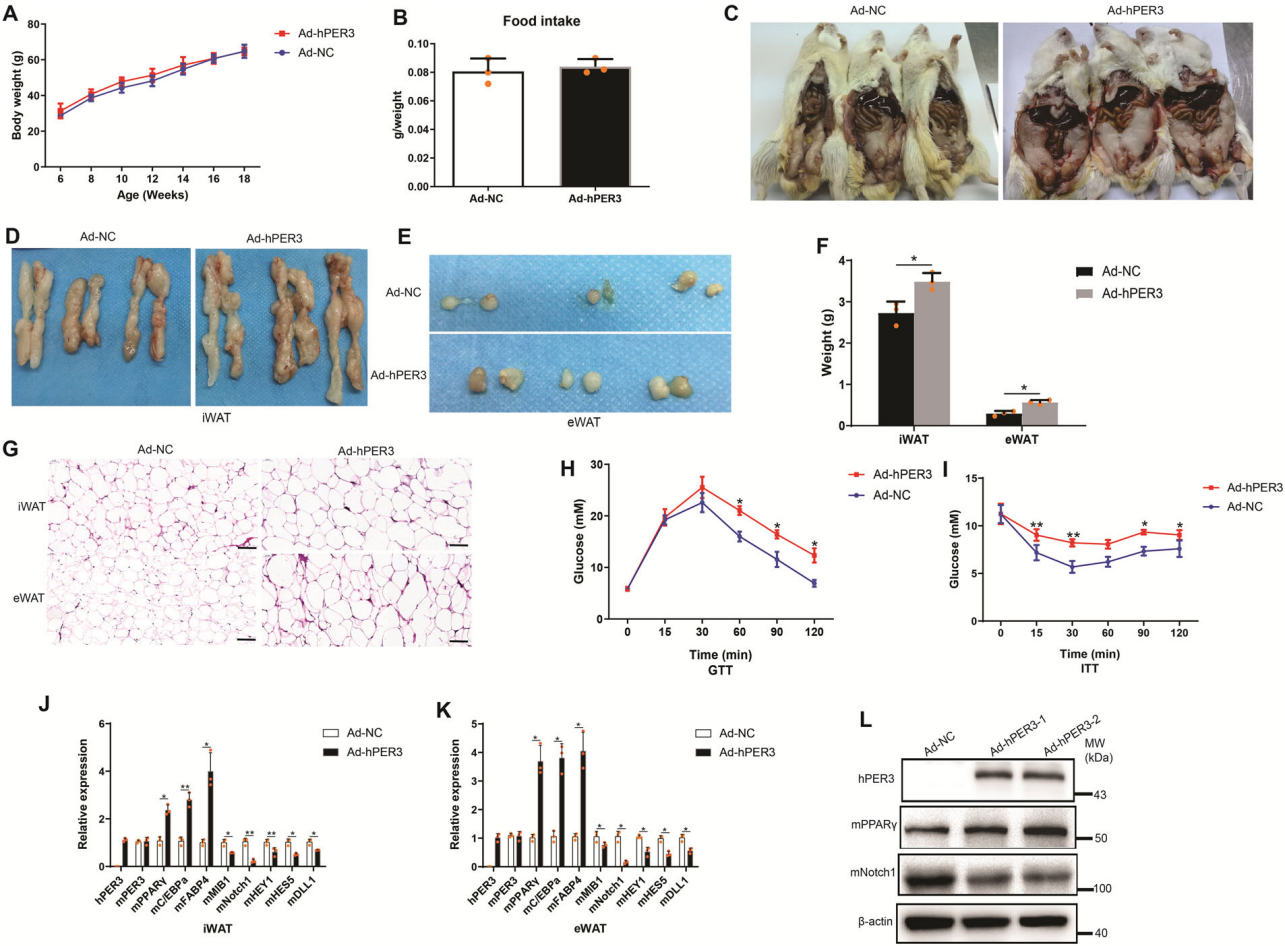
hPER3 increases adipogenesis in vivo by repressing mNotch1 pathway. Kunming mice were injected with Ad-NC and Ad-hPER3, and were subsequently fed with high-fat diet for 12 weeks. **A** Body weight after high-fat diet, *n* = 4. **B** Food intake after high-fat diet, *n* = 4. **C** iWAT, and **D** isolated iWAT and **E** isolated eWAT in vivo, *n* = 4. **F** The weight of iWAT and eWAT, *n* = 4. **G** H&E staining in iWAT and eWAT, scale bar = 100 μm, *n* = 4. **H** GTT and **I** ITT in Ad-NC and Ad-hPER3 groups, *n* = 4. **J** The mRNA expression of hPER3, mPER3, mPPARγ, mCEBP/α, mFABP4, and mNotch1 pathway in iWAT, *n* = 3. **K** Experimental settings are similar to **J**, the mRNA expression in eWAT, *n* = 3. **L** The protein-level expression of hPER3, mPPARγ, and mNotch1 in iWAT, *n* = 3. Data are presented as means ± SD. Student’s *t*-test, *p < 0.05, **p < 0.01.

To test whether hPER3 affects adipogenesis in vivo, Ad-NC and Ad-hPER3 were injected into the Kunming mice and were subsequently fed by high-fat diet for 12 weeks. Our results showed that although body weight (Fig. 6A) and food intake (Fig. 6B) showed no change between Ad-NC and Ad-hPER3 groups, the weight of iWAT and eWAT were increased in both the groups (Fig. 6C–F). In addition, hematoxylin and eosin (H&E) staining showed that the cell size in Ad-hPER3 group was larger than that of Ad-NC in iWAT and eWAT (Fig. 6G). Next, the effects of hPER3 on glucose metabolism after high-fat diet were evaluated and the results showed that hPER3 decreased glucose tolerance in the Ad-hPER3 group when compared to Ad-NC group (Fig. 6H). Furthermore, an insulin tolerance test (ITT) revealed a decreased response to exogenous insulin in Ad-hPER3 mice (Fig. 6I), indicating that increased hPER3 expression aggravated the development of high-fat diet-induced insulin resistance.

To further explore whether hPER3 regulates mNotch1 signaling pathway in vivo, the mRNA expression in iWAT and eWAT of Ad-NC and Ad-hPER3 groups was detected; among them, a specific hPER3 primer without mPER3 amplification was designed. Both in iWAT and eWAT, our results revealed that although mPER3 showed no change in the Ad-NC and Ad-hPER3 groups, hPER3 was markedly upregulated in the Ad-hPER3 group but hPER3 was undetected in the Ad-NC group. Nevertheless, the markers of mature adipocytes, both in iWAT and eWAT, including mPPARγ, mCEBP/α, and mFABP4 showed a significant increase in the Ad-hPER3 group, whereas the mNotch1 signaling pathway including mMIB1, mNotch1, mHEY1, mHES5, and mDLL1 were significantly downregulated (Fig. 6J, K). The protein-level expression was detected using western blotting and specific hPER3 antibody that does not detect mPER3 showed a strong increase in iWAT of mice in the Ad-hPER3 group, but hPER3 was completely undetected in the Ad-NC group, and drastically increased mPPARγ and decreased mNotch1 in iWAT (Fig. 6L). Due to very few eWAT samples, the protein levels were not tested in eWAT. Our results elucidated that hPER3 promoted adipogenesis in vivo and increased metabolic dysfunction via the hNotch1 pathway.

## Discussion

Circadian rhythm regulation of energy metabolism including glucose, lipid, food intake, hormone secretion, etc. has been extensively reported^2,34,35^, but different studies showed contradictory results. Knockout of ARNTL increased adipogenesis in mice^36^, whereas three studies showed a positive regulation of ARNTL in adipogenesis^37–39^. CLOCK also showed contradictory results and knockdown of CLOCK promoted early adipogenesis in 3T3-L1 cells and C57BL/6 mice adipose formation^40,41^; however, opposite results were observed in the study conducted by Boucher et al.^7^. Previous studies have shown that PER3 negatively or positively regulated adipogenesis in mouse and negatively regulated in humans^9–11,13^, and hPER3 in our study positively regulated adipogenesis in hADSCs.

Some studies have reported the association of hPER3 with obesity, for instance, PER3 methylation levels are related to childhood obesity^42^, hPER3 increased the BMI of those who sleep too late^13^, rs228669 of hPER3 showed association with triglycerides^43^, and cg10059324 also showed correlation with insulin resistance^44^. In our experiment, PER3 in humans or mice showed high expression in adipose tissues than in other tissues. Although Archer et al.^45^ have shown the highest PER3 expression in human retina and mice salivary and lacrimal glands using BioGPS GeneAtlas (biogps.org), these results provide evidence that PER3 is actually an important regulatory factor in adipose tissue. While different hADSCs isolated from four patients’ SATs showed that both mRNA and protein levels of hPER3 were gradually upregulated during adipogenesis, suggesting that hPER3 might positively regulate adipogenesis, Knarr et al.^12^ have reported that the expression of hPER3 was gradually downregulated during iBMSCs and PASC adipogenesis. This is because PASC was isolated from patient’s VATs and whether hPER3 plays different role in adipogenesis in VATs or bone marrow-derived MSCs still remained unclear.

Next, whether elevated or decreased hPER3 can affect adipogenesis was explored further and our results robustly supported the significance of hPER3 in promoting adipogenesis in hADSCs that were isolated from SATs, but three studies showed that mPER3 negatively regulated adipogenesis^9–11^ and two studies in humans have proved that hPER3 repressed adipogenesis^9,11^. Next, PER3 protein sequences in different animals were compared, and PER2 and PER3 showed significant differences in humans when compared to other animals including *M. musculus* and *R. norvegicus*, but other circadian rhythm regulation genes, such as *PER1*, *CLOCK*, *ARNTL*, *CRY1*, and *CRY2*, are highly conserved in others animal. Thus, although PER2 played opposite results in adipogenesis in the previous studies, Boucher et al.^7,8^ have proved that hPER2 positively regulated adipogenesis in hBMSCs, but mPER2 negatively regulated adipogenesis in MEFs and 3T3-L1 preadipocytes. Hence, we speculated that hPER3 also might play a different role in humans and other species as that of PER2.

Previous studies have elucidated that partial circadian genes indirectly combines with the promoter of PPARγ, e.g., mPER2 negatively regulates PPARγ by targeting S112^8^, Nocturnin promotes adipogenesis by stimulating PPARγ nuclear translocation, and Rev-erbα prevents adipogenesis by repressing PPARγ2^46–48^. What’s more, mPER3 inhibited PPARγ transcription activation by combining with PPAR response elements of PPARγ protein^9^. In addition, CLOCK was also proved to regulate transactivation of PPARα^49,50^. In our results, hPER3 significantly and positively regulated hPPARγ but not hSREBF1 and hCEBP/α in the four types of human cells. These results were completely opposite with that of mPER3 in the negative regulation of mPPARγ. Next, Co-IP results revealed that hPER3 and hPPARγ proteins had no interaction, speculating that hPER3 might regulate hPPARγ via an indirect pathway.

To further investigate the mechanism of hPER3 regulation of hPPARγ, RNA-seq was performed to find differential gene expression after silencing hPER3 in hADSCs. Our results showed differences in the expression of genes and were extensively enriched in Notch1 signaling pathway, subsequently proving that the overexpression of hPER3 decreased Notch1 signaling pathway. Although Notch signaling pathway is an evolutionarily conserved system^51–53^, Notch pathway in adipogenesis exhibited some controversial results. To further confirm the role of hNotch1 pathway in hADSCs adipogenesis, our results also showed that the gene expression of hNotch1 pathway was gradually downregulated during adipogenesis and silencing of hNotch1 significantly induced adipogenesis and upregulation of hPPARγ.

To further elucidate the mechanism of hPER3 in the regulation of hNotch1 pathway, overlapping genes from Co-IP + MS analysis and HuProt™ protein microarray were used to find the possible proteins that interact with hPER3 and combine with hNotch1 promoter. After verification by dual-luciferase reporter gene assay and Co-IP experiment, hHSP90AA1 was found to combine with hPER3 and hNotch1 promoter. HSP90AA1 participates in the regulation of PI3K/AKT/mTOR, aryl hydrocarbon receptor pathway, etc.^54,55^, but it did not participate in the adipogenesis or metabolism and Notch pathway. Kim et al.^56^ reported that HSP90AA1 showed no change during 3T3-L1 adipogenesis and this indicated that hHSP90AA1 plays a bridging role between hPER3 and Notch1 pathway.

In knockout mPER3 model, increased body weight was observed only in male but not in female mice. In male mice, SATs and perigonadal fat were significantly upregulated, but perigonadal fat was increased in female mice when compared to controls^10^. In vivo experiments showed that the fat mass was re-distributed after overexpressing hPER3 in mice and the adipose weight of iWAT and eWAT were increased. In addition, the expression of mNotch pathway might be affected by hPER3. Our results elucidated that hPER3 could promote adipogenesis and induce glucose dysfunction and insulin insistence.

Taken together, our results showed hPER3 as a positive regulatory factor that participates in adipogenesis, although our study showed opposite results with that of the previous studies. Our findings provided a new understanding with regard to hPER3 regulation of adipogenic differentiation and metabolism.

## Materials and methods

### Patients and sample collection

The SATs and VATs in the omentum majus from normal patients (BMI: 18.5–23.9, *n* = 8, male = 4, female = 4), lean (BMI: < 18.5, *n* = 8, male = 4, female = 4), and obese (BMI: ≥ 32, *n* = 8, male = 4, female = 4) individuals were collected. In addition, other tissues samples including the pancreas, liver, small intestine, and stomach, from four unrelated patients with no diabetes, acute inflammation, and cancer were selected and recruited from the Department of General Surgery, The Third Xiangya Hospital of Central South University (Changsha, China). All the tissue samples were collected through surgical method and immediately preserved at −80 °C until analysis. This study was approved by the Ethics Committee of The Third Xiangya Hospital of Central South University, Changsha, China (number: 2018-S110), and was performed according to the guidelines outlined in the Declaration of Helsinki. All participants provided written informed consent form.

### High-fat diet and sample collection in mice

C57BL/6 male mice (4 weeks, *n* = 8) were fed on Chow diet for 2 weeks and then four of eight mice were fed with high-fat diet (60% fat, 20% carbohydrates, and 20% proteins) (D12492, Research Diets, New Brunswick, NJ), and the remaining four mice continued Chow diet for 12 weeks. Next, the iWAT and the eWAT were collected. In addition, C57BL/6 (4 weeks, *n* = 4) were fed by Chow diet for 4 weeks and then the tissue samples including the iWAT, eWAT, liver, pancreas, etc. were collected to explore the expression of mPER3 mRNA in different tissues. In addition, the iWAT were isolated from 21 C57BL/6 mice (6 weeks, *n* = 3) to detect the mPER3 expression during 24 h circadian time in every 4 h. All mice samples were collected at 8 h of circadian time. All tissue samples were preserved at −80 °C until analysis. All experiments were performed according to the guidelines of the Ethical Committee for Animal Experiments of The Third Xiangya Hospital of Central South University (Changsha, China).

### Isolation, differentiation, Oil Red O staining, and absorbance test of hADSCs or mADSCs

The isolation, differentiation, and Oil Red O staining of hADSCs or mADSCs were conducted as described in our previous study^34^. Briefly, 12 g of fresh abdominal SATs were isolated from four normal BMI (18.5–23.9) patients and iWAT was isolated from C57BL/6 male mice (*n* = 4). Adipose tissues were washed in phosphate buffer saline (PBS) three times and the tissue was subsequently cut into 1 mm × 1 mm size, followed by incubation in collagenase I solution (Gibco, Life Technology, China) for 1.5 h at 37 °C. The digested tissue mixture was filtered using a 70 µm cell strainer (BD Falcon, Becton Dickinson, Franklin Lakes, NJ, USA), centrifuged at 150 × *g* for 10 min and then the supernatant was poured off. Erythrocyte lysate (3 ml; Beyotime Institute of Biotechnology, Shanghai, China) was added into the sample for 3 min at room temperature, centrifuged at 150 × *g* for 10 min again, and the precipitate was washed by PBS thrice. Finally, the sample was cultured in Dulbecco’s modified Eagle medium/F12 (Life Technologies, Carlsbad, CA, USA) and supplemented with 10% fetal bovine serum (Life Technologies). The cellular lipid droplets content was extracted by 100% isopropanol and absorbance quantification was detected by using a spectrometer at 520 nM (Multiskan FC; Thermo Fisher Scientific, Inc., Waltham, MA, USA).

### RNA preparation and qRT-PCR

Total RNA was extracted from cells or tissues by TRIzol reagent (Life Technologies, Carlsbad, CA, USA). Next, cDNA synthesis was performed using a reverse-transcription kit (Promega, Madison, WI, USA). RT-qPCR was used by a Mastercycler^®^ real-time PCR (Eppendorf, Hamburg, Germany) and gene primers were provided in Supplementary Table 2. The relative mRNA expression was calculated by 2^−ΔΔCT^ method and the values were normalized to the expression of glyceraldehyde 3-phosphate dehydrogenase. The experiment was duplicated and repeated three times.

### Western blotting

The tissues or cells were lysed in a radioimmunoprecipitation assay buffer (Sigma-Aldrich, St Louis, MO, USA) and the protein concentration was quantified by using bicinchoninic acid assay (Beyotime Institute of Biotechnology, Shanghai, China). The following primary antibodies were used: special hPER3 (sc-517227, 1 : 200, Santa Cruz Biotechnology), PER1 (13463-1-AP, 1 : 300, proteintech), PER2 (20359-1-AP, 1 : 500, proteintech), PPARγ (ab45036, 1 : 600, Abcam), CLOCK (18094-1-AP, 1 : 400, proteintech), ARNTL (14268-1-AP, 1 : 300, proteintech), CRY1 (13474-1-AP, 1 : 500, proteintech), CRY2 (13997-1-AP, 1 : 400, proteintech), Notch1 (20687-1-AP, 1 : 500, proteintech), HES5 (22666-1-AP, 1 : 500, proteintech), HEY1 (19929-1-AP, 1 : 1000, proteintech), DLL1 (20230-1-AP, 1 : 500, proteintech), MIB1 (11893-1-AP, 1 : 500, proteintech), Flag (F1804, 1 : 2000, Sigma), hHSP90AA1 (ab2928, 1 : 400, Abcam), and β-actin (66009-1-Ig, 1 : 10,000, proteintech). The experiment was duplicated and repeated three times.

### Ad-hPER3 construction and transfection

The full-length hPER3 (NM_001289861.2) open reading frame was amplified by a specific primer, forward: 3′-AGGTCGACTCTAGAGGATCCCGCCACCATGCCCCGCGGGGAAGCTCCTGGCCCCGGGAGAC-5′, and reverse: 3′-TCCTTGTAGTCCATACCACAGCTGTCTTCTACCAGAACCTGACCAC-5′. The product was inserted into the GV314 adenoviral system (GeneChem, Shanghai, China) by using BamHI/AgeI sites and was subsequently packed into an adenovirus. The negative control virus (Ad-NC) was purchased from Genechem and all adenovirus was stored at −80 °C. The transfection method including the screening of the optimal transfection concentration was performed according to manufacturer’s instructions and, after cell transfection for 72 h, the infection efficiency was verified under a fluorescence microscope (Leica DM IL LED Fluo, Germany) and by western blotting.

### LV-shRNA-hPER3, LV-shRNA-hNotch1, and transfection

LV-shRNA-hPER3 (sc-38173-V), LV-shRNA-hNotch1 (sc-36095-V), and LV-shRNA-NC (sc-108080) were purchased from Santa Cruz Biotechnology. The transfection method was performed according to the manufacturer’s instructions. Briefly, 1 × 105 hADSCs or 293 cells were cultured in six-well plates for 24 h later, transfected with lentiviral plasmid in a serum-free medium for 16 h, and then changed with a complete medium for 72 h. The silencing efficiency of target genes was detected by western blotting and the plasmid with the highest knockdown efficiency was chosen for subsequent experiments.

### Immunofluorescence analysis

After hADSCs were transfected with Ad-hPER3, Ad-NC, LV-shRNA-hPER3, and LV-shRNA-NC for 72 h, induced adipocytes, and then were collected on day 12. The cells were fixed by 4% paraformaldehyde and washed with PBS three times, blocked, and permeabilized with 0.5% Triton X-100 (Sigma, St. Louis, MO, USA) and 1% bovine serum albumin-supplemented PBS at room temperature for 20 min, washed with PBS thrice again, and 5% normal goat serum containing 0.1% Triton X-100 in PBS was used to block the antibody. The cells were incubated with primary PLIN1 antibody (#9349, 1 : 100, CST) overnight at 4 °C and labeled using an Alexa Fluor-conjugated secondary antibody (1 : 500 dilution; Invitrogen, Thermo Fisher Scientific, Waltham, MA, USA). The reaction was developed using avidin-Texas Red-conjugated (A-2006, Vector Laboratories, 1 : 1000) and the nuclei was stained with DAPI (Sigma-Aldrich, USA). Lastly, the images were captured with a fluorescence microscope (Leica DM IL LED Fluo, Germany). The experiment was duplicated and repeated three times.

### RNA-seq and data analysis

The hADSCs were transfected with LV-shRNA-hPER3 or LV-shRNA-NC for 72 h, the cells were collected, RNA was extracted by TRizol reagent, and the quality of RNA was detected using RNA Nano 6000 Assay Kit of the Bioanalyzer 2100 system (Agilent Technologies, Santa Clara, CA, USA). A cDNA library was constructed using a NEB Next^®^ Ultra™ RNA Library Prep Kit for Illumina^®^ (New England Biolabs, Ipswich, MA, USA) according to the manufacturer’s protocol. The data were produced by Illumina Hiseq 2000/2500 platform and the differential gene expression was identified using the DESeq R package from http://bioinfo.au.tsinghua.edu.cn/software/degseq. The GO enrichment was analyzed with GOseq R package and pathway enrichment was analyzed with the Kyoto Encyclopedia of Genes and Genomes (KEGG) database (www.genome.jp/kegg) and KOBAS software.

### hHSP90AA1, hHNRNPK, and hPPARγ plasmid construction

The molecular clone protocol was done according to the previous study^35^. The full-length of human HSP90AA1 (NM_001017963.3), hHNRNPK (NM_002140.5), and hPPARγ (NM_015869.5) open reading frames were amplified and connected with flag label (hHSP90AA1-Flag, hHNRNPK-Flag and hPPARγ-Flag) and then inserted into GV141 vector (GeneChem, Shanghai, China). The primer sequences of hHSP90AA1 were as follows: forward: 3′-ACGGGCCCTCTAGACTCGAGCGCCACCATGCCCCCGTGTTCGGGCGGGGACGGC-5′, reverse: 3′-AGTCACTTAAGCTTGGTACCGAGTCTACTTCTTCCATGCGTGATGTGTC-5′; hHNRNPK, forward: 3′-ACGGGCCCTCTAGACTCGAGCGCCACCATGGAAACTGAACAGCCAGAAG-5′, reverse: 3′-AGTCACTTAAGCTTGGTACCGAGAATCCTTCAACATCTGCATACTGCTTCACACTG-5′; hPPARγ, forward: 3′-ACGGGCCCTCTAGACTCGAGCGCCACCATGGGTGAAACTCTGGGAGATTC-5′, reverse: 3′-AGTCACTTAAGCTTGGTACCGAGTACAAGTCCTTGTAGATCTCCTGC-5′. The product was cut with XhoI/KpnI (New England BioLabs, Inc., Ipswich, MA, USA). The full-length of hHSP90AA1 was inserted into GV314 vector and used to construct the Lentiviral hHSP90AA1 (LV-hHSP90AA1) plasmid.

### Co-immunoprecipitation

The 293 cells were transfected with hHSP90AA1-flag or hHNRNPK-flag, or hPPARγ-flag plasmid for 72 h, respectively. The cells were then lysed in a Co-IP buffer (20 mM Tris pH 7.5, 150 mM NaCl, and 1% Triton X-100; P0013, BBI, Shanghai, China) and then centrifuged at 14,000 × *g* for 15 min at 4 °C. After precleared with protein A/G-agarose, the supernatants were incubated with anti-FLAG^®^ M2 affinity Gel (Sigma, A2220, 1 : 2000) for overnight. Next, the samples were continuously incubated with protein A/G-agarose at 4 °C for 4 h. The cold lysis buffer was washed thrice, added sodium dodecyl sulfate loading buffer, and heated to 98 °C for 10 min. Protein expression was then performed according to the western blotting protocol.

### Co-IP + MS analysis

The Co-IP was performed as described above. The MS was performed by BiotechPack Technology Company, Ltd (Beijing, China). Briefly, 10 μL trypsin (Promega V5280, 15 ng/μL diluted with 25 mmol/L NH_4_HCO_3_) was added and incubated at 4 °C for 40 min, followed by enzyme digestion at 37 °C for 16 h and addition of 100 μL of 0.5% trifluoroacetic acid (TFA) and 50% acetonitrile (ACN) for 1 h at 37 °C. After centrifugation, 100 μL of 0.5% TFA and 50% ACN were added at 37 °C for 1 h again and then the supernatant was vacuum-dried. The peptide was resolved with 0.1% formic acid and 2% TFA to immediately detect the results with Q Exactive mass spectrometer (Thermo Fisher), and then the raw data were analyzed using Mascot software.

### Promoter of hNotch1 plasmid construction and HuProt™ protein microarray

About 2000 bp promoter sequence plasmid of hNotch1 was constructed. Briefly, 2000 bp promoter sequence of hNotch1 (NM_017617.5) was amplified using special primer, forward: 3′-GATCCAGTTTGGTTAATTAAGACCAGGGGAGACCCCCTATCC-5′; reverse: 3′-GTACCCGGGGATCCTCTAGAGCCTCCCCACCGGCTGCCCTC-5′. The product was cut with PacI/XbaI (New England BioLabs, Inc., Ipswich, MA, USA) and then inserted into GV341 vector (GeneChem, Shanghai, China). To identity the possible combination proteins with the promoter of hNotch1, HuProt™ microarray (HuProtTM, Cambridge Protein Arrays) was conducted according to the manufacturer’s instructions. Briefly, the protein microarray was incubated by blocking buffer (25 mM HEPES pH 8.0, 50 mM KCl, 8 mM MgCl_2_, 4 μM poly (dA-dT), 3 mM dithiothreitol (DTT), 0.1% Triton X-100, 10% glycerol) at 4 °C for 3 h, removed the blocking buffer, and 11 nM hNotch1 plasmid containing 2000 bp promoter sequence was diluted with incubation solution (25 mM HEPES pH 8.0, 50 mM KCl, 8 mM MgCl_2_, 4 μM poly (dA-dT), 3 mM DTT, 0.1% Triton X-100, 10% glycerol) at 4 °C overnight. After that, the sample was washed with washing solution (25 mM HEPES pH 8.0, 50 mM KCl, 8 mM MgCl_2_, 3 mM DTT, 0.1% Triton X-100, 10% glycerol) three times and washed with ddH_2_O twice. The microarray was dried with SlideWasher 120090 (CapitalBio Technology, Inc. Beijing, China) and the data were scanned using LuxscanTM 10Κ-A scanner (CapitalBio Technology, Inc. Beijing, China) and GenePix Pro v6.0. The probes were detectable when the SNRs for both duplicates were over 1.3. The GO (http://geneontology.org/), KEGG (https://www.genome.jp/kegg/pathway.html), and String (http://string-db.org/cgi/input.pl) databases were used for GO, pathway, and protein interaction analysis, respectively.

### Dual-luciferase reporter gene assay

For obtaining possible proteins that can interact with hPER3 and combine with the promoter of hNotch1, 23 proteins associated with adipogenesis and overlapping data in Co-IP + MS and HuProt™ protein microarray were chosen. To further verify these results, whether it can combine with the promoter of hNotch1, a dual-luciferase reporter gene assay was used. The full-length open reading frame of these genes was amplified by a specific primer (Supplementary Table 3) and digested with XhoI/KpnI (New England BioLabs, Inc., Ipswich, MA, USA). The target DNA fragment was cloned into a GV230 (Genechem, Shanghai, China) using DNA ligation kit (Takara Bio, Inc., Dalian, China). NC was an empty GV230-basic vector and the positive control was a GV230-basic vector containing the upstream of the SV40 promoter. The 293 cells were used for dual-luciferase reporter gene assay (E2910, Promega, Madison, WI).

### Mice tail vein injection

After the male Kunming mice (4 weeks, *n* = 8) were fed with Chow diet for 2 weeks, 200 μL (2 × 10^10^ PFU/ml) Ad-NC (*n* = 4) or Ad-hPER3 (*n* = 4) was injected into the tail vein once a week and the remaining mice were fed with high-fat diet for 12 weeks, respectively.

### GTT and ITT tests

After the Kunming mice were transfected with Ad-NC or Ad-hPER3 and fed high-fat diet for 12 weeks, glucose tolerance test (GTT) and ITT were performed. In GTT, the mice were fasted for 16 h and the blood glucose was measured using a glucometer (Roche) at 0, 15, 30, 45, 60, 90, and 120 min via tail after injecting 1.5 g/kg glucose intraperitoneally. For ITT, the mice were fasted for 6 h, 1 U/kg recombinant human insulin (Novo Nordisk, China) was injected intraperitoneally, and the blood glucose levels were detected at 0, 15, 30, 45, 60, 90, and 120 min.

### H&E staining

The iWAT and eWAT were collected and fixed in 4% paraformaldehyde, embedded in paraffin for the preparation of 4 µm-thick tissue sections, the slices were stained with H&E, and then the images were obtained using fluorescence microscope (Leica DM IL LED Fluo, Germany).

### Statistical analysis

The results were summarized and presented as means ± SD and were analyzed using Student’s *t*-test for the two groups, and multiple comparisons were carried out using analysis of variance and were further carried out for post hoc analysis. The statistically significant level was defined as *p* < 0.05.

## Supplementary information

Supplementary figure legends

supplementary fig1

supplementary fig2

supplementary fig3

supplementary fig4

Supplementary Table 1

Supplementary Table 2 Primer sequences used for qRT-PCR

Supplementary Table 3
