# Supplementary material for: hPER3 promotes adipogenesis via hHSP90AA1-mediated inhibition of Notch1 pathway
Source: Cell Death Dis. 2021 Mar 19;12(4):301. doi: 10.1038/s41419-021-03584-0 (PMC7979882; doi:10.1038/s41419-021-03584-0)
Supplement: Supplementary file 1 — Supplementary figure legends [file 41419_2021_3584_MOESM1_ESM.docx]

**Supplementary Figure Legends**

**Supplementary Fig. S1 The partial circadian rhythm genes were gradually upregulated during hADSCs adipogenesis.** (A) The mPER3 mRNA of 24 hours circadian rhythm level in C57BL/6 iWAT were detected, n = 3. (B to E) represent the mRNA expression of hCLOCK, hARNTL, hCRY1 and hCRY2 during adipogenesis, n = 4, the relative expression was compared to day 0. Data are presented as means ± SD. Paired two-tailed Student′s t-test, ***p < 0.01*, ****p < 0.001*, and *****P < 0.0001*.

**Supplementary Fig. S2 hPER3 does not effect on hCEBP/α and hSREBF1.** (A, B) The mRNA expression of hCEBP/α and hSREBF1 after hADSCs was transfected with Ad-NC, Ad-hPER3, LV-shRNA-NC and LV-shRNA-hPER3 for 72h, n = 3. Data are presented as means ± SD.

**Supplementary Fig. S3 The transcription profile of silencing hPER3 in hADSCs.** (A) The heatmap represents transcription profiles after knockdown of hADSCs by hPER3. n = 3. (B) Go analysis showing enrichment of differential genes, and the plot size represents gene enrichment numbers, the color from red to purple represents the significance of enrichment. (C, D) The translation efficiency of LV-shRNA-hNotch1 was evaluated by RT-qPCR (C) and Western blotting (D). (E) The hHSP90AA1 mRNA expression during adipogenesis, n = 4. Data are presented as means ± SD. Paired two-tailed Student′s t-test, ***p < 0.01*, ****p < 0.001*, and *****P < 0.0001*.

**Supplementary Fig. S4 hHSP90AA1 does not interact with hHNRNPK.** The interaction of hHSP90AA1 and hHNRNPK was detected by CO-IP, n = 3.
