# Supplementary figures and images for: hPER3 promotes adipogenesis via hHSP90AA1-mediated inhibition of Notch1 pathway
Source: Cell Death Dis. 2021 Mar 19;12(4):301. doi: 10.1038/s41419-021-03584-0 (PMC7979882; doi:10.1038/s41419-021-03584-0)

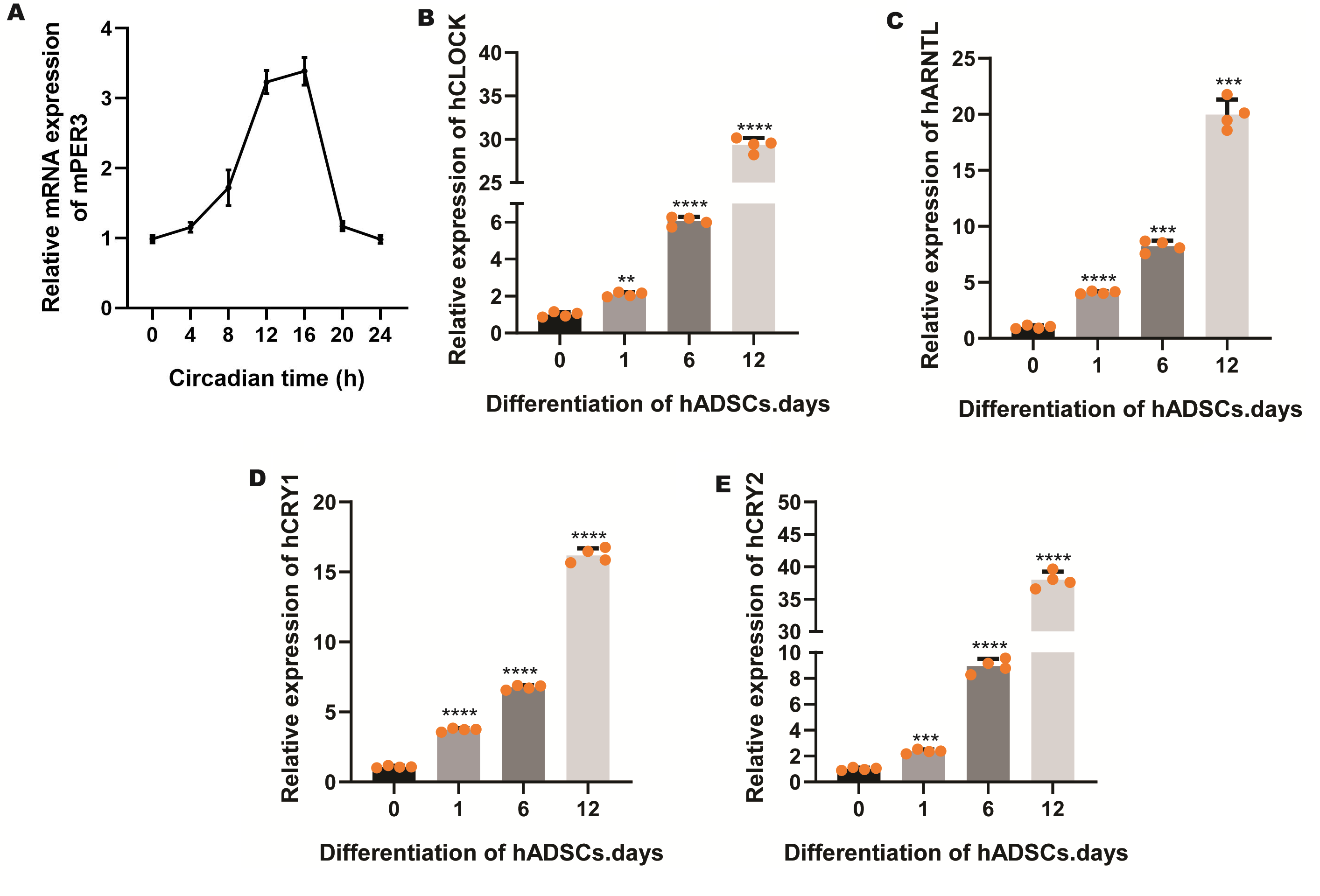

Supplement: Supplementary file 2 — supplementary fig1 [file 41419_2021_3584_MOESM2_ESM.tif]

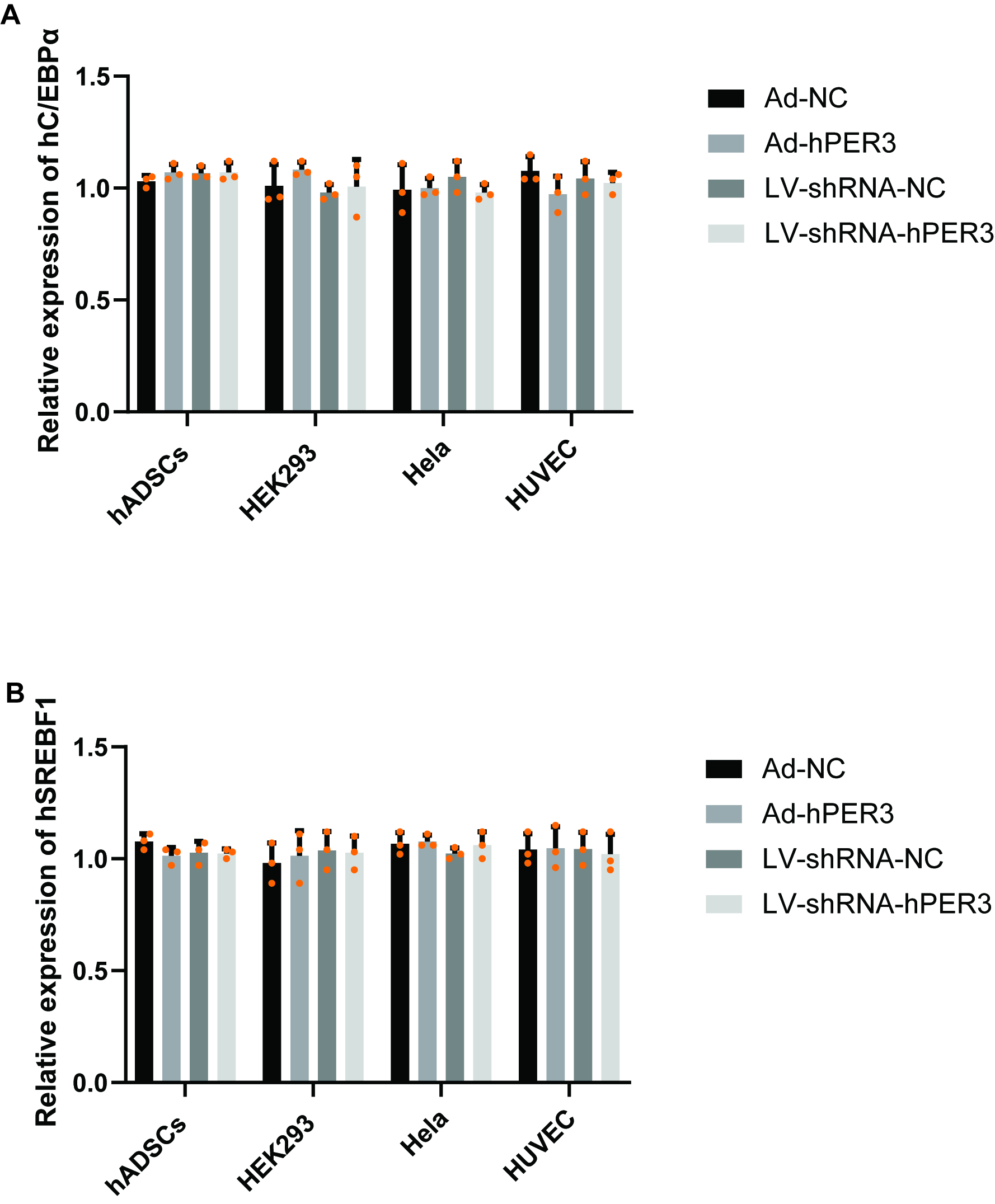

Supplement: Supplementary file 3 — supplementary fig2 [file 41419_2021_3584_MOESM3_ESM.tif]

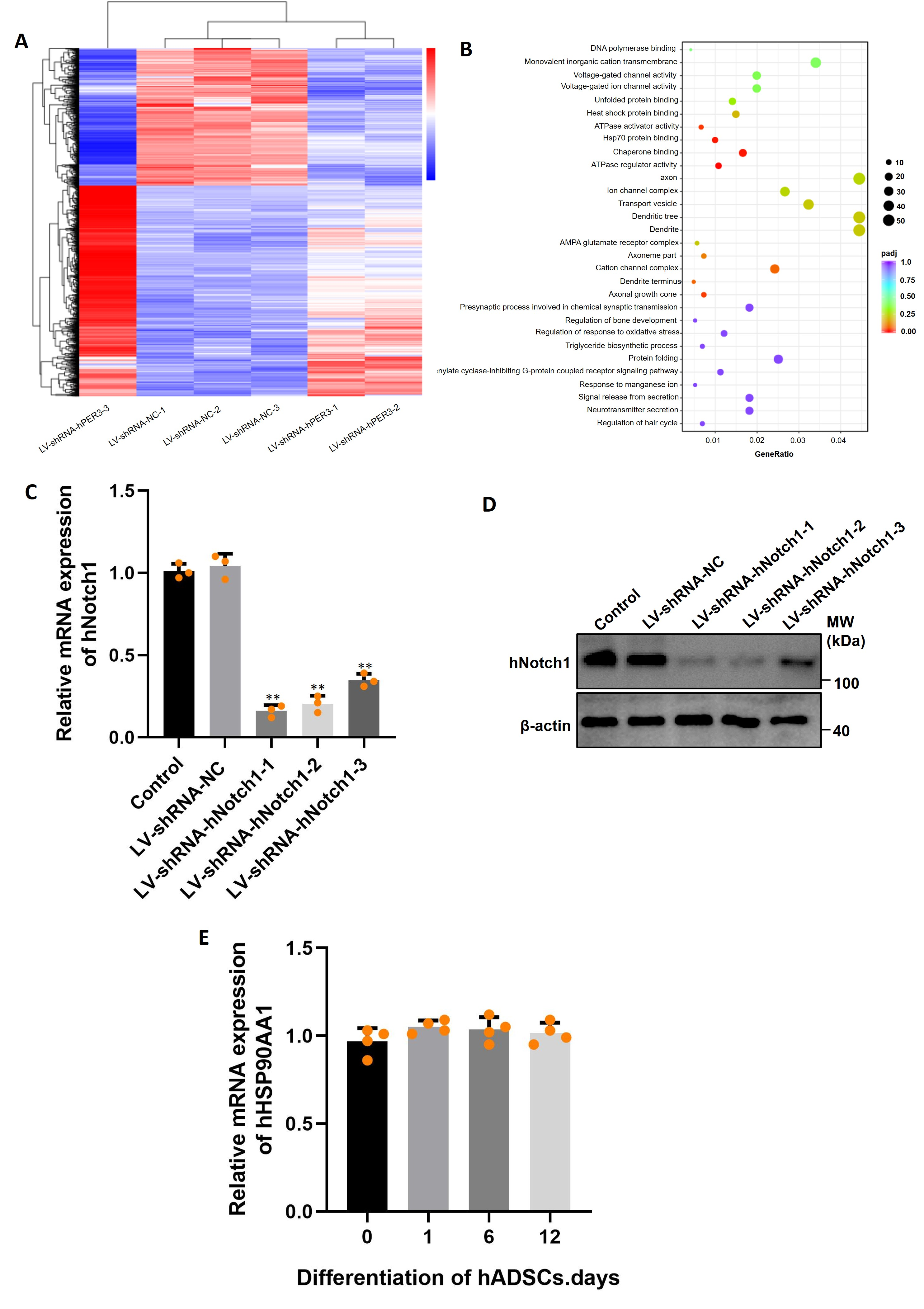

Supplement: Supplementary file 4 — supplementary fig3 [file 41419_2021_3584_MOESM4_ESM.tif]

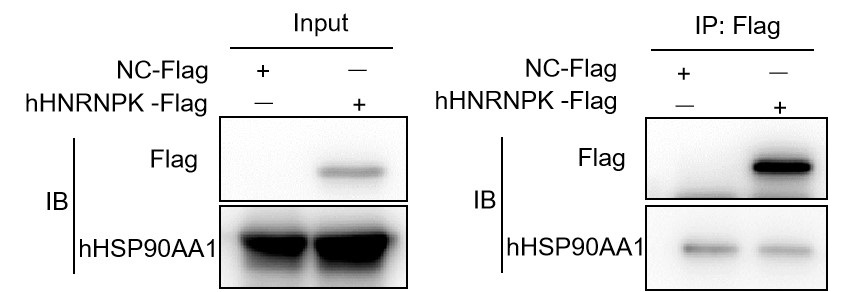

Supplement: Supplementary file 5 — supplementary fig4 [file 41419_2021_3584_MOESM5_ESM.tif]
