# Supplementary material for: hPER3 promotes adipogenesis via hHSP90AA1-mediated inhibition of Notch1 pathway
Source: Cell Death Dis. 2021 Mar 19;12(4):301. doi: 10.1038/s41419-021-03584-0 (PMC7979882; doi:10.1038/s41419-021-03584-0)
Supplement: Supplementary file 6 — Supplementary Table 1 [file 41419_2021_3584_MOESM6_ESM.docx]

**Supplementary Table 1 Different circadian rhythm protein sequences in fewer animals**

| **Species** | **PER3** | **PER2** | **PER1** | **ARNTL** | **CLOCK** | **CRY1** | **CRY2** |
| --- | --- | --- | --- | --- | --- | --- | --- |
| *Homo sapiens (human)* | 100.0% | 100.0% | 100.0% | 100.0% | 100.0% | 100.0% | 100.0% |
| *Pan troglodytes (chimpanzee)* | 98.3% | 99.8% | 99.8% | 99.8% | 99.9% | 99.6% | 99.8% |
| *Gorilla gorilla gorilla* *(western lowland gorilla)* | 97.7% | / | / | / | 99.8% | 100.0% | 99.6% |
| *Hylobates moloch (silvery gibbon)* | 95.4% | 96.3% | 97.7% | 97.7% | 99.5% | 100.0% | 99.6% |
| *Pongo abelii (Sumatran orangutan)* | 94.6% | 98.3% | 98.1% | 99.7% | 99.9% | 99.5% | 99.6% |
| *Nomascus leucogenys* *(northern white-cheeked gibbon)* | 94.2% | 97.9% | 98.0% | 99.8% | 99.8% | 100.0% | 99.4% |
| *Equus caballus (horse)* | 73.5% | 78.5% | 96.0% | 96.0% | 99.3% | 93.4% | 97.5% |
| *Bos taurus (cattle)* | 67.3% | 73.0% | 89.9% | 89.9% | 96.9% | 98.1% | 95.7% |
| *Rattus norvegicus (Norway rat)* | 65.9% | 77.1% | 90.8% | 90.8% | 88.7% | 96.3% | 95.6% |
| *Mus musculus (house mouse)* | 65.7% | 77.4% | 91.7% | 91.7% | 93.6% | 93.4% | 96.0% |
| *Canis lupus familiaris (dog)* | 64.0% | 73.2% | 91.6% | 91.6% | 94.0% | 98.5% | 97.7% |
| *Sus scrofa (pig)* | 59.5% | 75.3% | 91.3% | 91.3% | 88.9% | 98.0% | 97.7% |
| *Oryctolagus cuniculus (rabbit)* | 56.3% | 72.8% | 95.1% | 95.1% | 89.3% | 97.8% | 97.3% |
| *Gallus gallus (chicken)* | 52.3% | 58.4% | / | 93.1% | 85.3% | 87.0% | 88.3% |
