# Supplementary material for: hPER3 promotes adipogenesis via hHSP90AA1-mediated inhibition of Notch1 pathway
Source: Cell Death Dis. 2021 Mar 19;12(4):301. doi: 10.1038/s41419-021-03584-0 (PMC7979882; doi:10.1038/s41419-021-03584-0)
Supplement: Supplementary file 7 — Supplementary Table 2 Primer sequences used for qRT-PCR [file 41419_2021_3584_MOESM7_ESM.docx]

**Supplementary Table 2 Primer sequences used for qRT-PCR**

| **Gene name** | **Primer sequence (5’-3’)** |
| --- | --- |
| hPER3 | 5’-GTCCAAGCCTTACAAGCTGGTTT-3’ |
|  | 5’-GACCGTCCATTTGTTGGCAT-3’ |
| mPER3 | 5’-AACACGAAGACCGAAACAGAAT-3’ |
|  | 5’-CTCGGCTGGGAAATACTTTTTCA-3’ |
| hPER1 | 5’-ACGGGCCGAATCGTCTACA-3’ |
|  | 5’-TGGAACCATAGAAGACTCCCAC-3’ |
| mPER1 | 5’-TGAAGCAAGACCGGGAGAG -3’ |
|  | 5’-CACACACGCCGTCACATCA-3’ |
| hPER2 | 5’-GACATGAGACCAACGAAAACTGC-3’ |
|  | 5’-AGGCTAAAGGTATCTGGACTCTG-3’ |
| mPER2 | 5’-GAAAGCTGTCACCACCATAGAA -3’ |
|  | 5’-AACTCGCACTTCCTTTTCAGG-3’ |
| hCLOCK | 5’- TGCGAGGAACAATAGACCCAA-3’ |
|  | 5’-ATGGCCTATGTGTGCGTTGTA-3’ |
| hARNTL | 5’- AAGGGAAGCTCACAGTCAGAT-3’ |
|  | 5’-GGACATTGCGTTGCATGTTGG-3’ |
| hCYR1 | 5’-CTCCTCCAATGTGGGCATCAA-3’ |
|  | 5’-CCACGAATCACAAACAGACGG-3’ |
| hCYR2 | 5’-TCCCAAGGCTGTTCAAGGAAT -3’ |
|  | 5’-TGCATCCCGTTCTTTCCCAAA-3’ |
| hC/EBPα | 5’-TGGACAAGAACAGCAACGAG-3’ |
|  | 5’-TTGTCACTGGTCAGCTCCAG-3’ |
| mC/EBPα | 5’-CAAGAACAGCAACGAGTACCG-3’ |
|  | 5’-GTCACTGGTCAACTCCAGCAC-3’ |
| hPPARγ | 5’- GAGAAGACTCAGCTCTAC-3’ |
|  | 5’- CAAGCATGAACTCCATAGTG-3’ |
| mPPARγ | 5'-TCGCTGATGCACTGCCTATG-3’ |
|  | 5’-GAGAGGTCCACAGAGCTGATT-3’ |
| hSREBF1 | 5'-CGGAACCATCTTGGCAACAGT-3’ |
|  | 5’-CGCTTCTCAATGGCGTTGT-3’ |
| hFABP4 | 5’-AGCACCATAACCTTAGATGGGG-3’ |
|  | 5’- CGTGGAAGTGACGCCTTTCA-3’ |
| mFABP4 | 5’-AAGGTGAAGAGCATCATAACCCT-3’ |
|  | 5’-TCACGCCTTTCATAACACATTCC-3’ |
| hADIPOQ | 5’-TGCTGGGAGCTGTTCTACTG-3’ |
|  | 5’-TACTCCGGTTTCACCGATGTC-3’ |
| hMIB1 | 5’-ATCATTGGCATTCGATGGAAGT-3’ |
|  | 5’-CCCGGTGTAGTAATTCGGTAAAA-3’ |
| mMIB1 | 5’-AGTTGGCCGAGTACAACAGAT-3’ |
|  | 5’-TGTTCCACAGACTTCCACCTT-3’ |
| hNotch1 | 5’-GAGGCGTGGCAGACTATGC-3’ |
|  | 5’-CTTGTACTCCGTCAGCGTGA-3’ |
| mNotch1 | 5’- GATGGCCTCAATGGGTACAAG-3’ |
|  | 5’- TCGTTGTTGTTGATGTCACAGT-3’ |
| hHEY1 | 5’-GTTCGGCTCTAGGTTCCATGT -3’ |
|  | 5’-CGTCGGCGCTTCTCAATTATTC -3’ |
| mHEY1 | 5’-CCGACGAGACCGAATCAATAAC -3’ |
|  | 5’-TCAGGTGATCCACAGTCATCTG -3’ |
| hHES5 | 5’- AAGCACAGCAAAGCCTTCGT-3’ |
|  | 5’-TGGAGCGTCAGGAACTGCAC-3’ |
| mHES5 | 5’-AGTCCCAAGGAGAAAAACCGA -3’ |
|  | 5’-GCTGTGTTTCAGGTAGCTGAC-3’ |
| hDLL1 | 5’- GACGAACACTACTACGGAGAGG-3’ |
|  | 5’-AGCCAGGGTTGCACACTTT-3’ |
| mDLL1 | 5’-CAGGACCTTCTTTCGCGTATG -3’ |
|  | 5’-AAGGGGAATCGGATGGGGTT-3’ |
| hHSP90AA1 | 5’- CATAACGATGATGAGCAGTACGC-3’ |
|  | 5’- GACCCATAGGTTCACCTGTGT-3’ |
| hGAPDH | 5’-AGCCAGGGTTGCACACTTT-3’ |
|  | 5’-CAGCCTTCTCCATGGTGGTGAAGA-3’ |
| mGAPDH | 5’- AGGTCGGTGTGAACGGATTTG-3’ |
|  | 5’-TGTAGACCATGTAGTTGAGGTCA-3’ |
