# Supplementary material for: hPER3 promotes adipogenesis via hHSP90AA1-mediated inhibition of Notch1 pathway
Source: Cell Death Dis. 2021 Mar 19;12(4):301. doi: 10.1038/s41419-021-03584-0 (PMC7979882; doi:10.1038/s41419-021-03584-0)
Supplement: Supplementary file 8 — Supplementary Table 3 [file 41419_2021_3584_MOESM8_ESM.docx]

**Supplementary Table 3 Primer sequences used for PCR amplification of dual luciferase reporter genes**

| **Gene name** | **Accession** | | **Primer sequence (5’-3’)** |
| --- | --- | --- | --- |
| IGFBP1 | | NM_000596.4 | 5’-TACCGGACTCAGATCTCGAGCGCCACCATGTCAGAGGTCCCCGTTGC-3’ |
|  |  |  | 5’-GATCCCGGGCCCGCGGTACCGTGTTTTGTACATTAAAATATATCTG-3’ |
| NCL | | NM_005381.3 | 5’-TACCGGACTCAGATCTCGAGCGCCACCATGGTGAAGCTCGCGAAGGC-3’ |
|  |  |  | 5’-GATCCCGGGCCCGCGGTACCGTTTCAAACTTCGTCTTCTTTCCTTGTGG-3’ |
| UNG | | NM_080911.3 | 5’-TACCGGACTCAGATCTCGAGCGCCACCATGATCGGCCAGAAGACGCTC-3’ |
|  |  |  | 5’-GATCCCGGGCCCGCGGTACCGTCAGCTCCTTCCAGTCAATGGGCTTC-3’ |
| HNRNPK | | NM_002140.5 | 5’-TACCGGACTCAGATCTCGAGCGCCACCATGGAAACTGAACAGCCAGAAGAAAC-3’ |
|  |  |  | 5’-GATCCCGGGCCCGCGGTACCGTGAATCCTTCAACATCTGCATACTGC-3’ |
| YBX2 | | NM_015982.4 | 5’-TACCGGACTCAGATCTCGAGCGCCACCATGAGCGAGGTGGAGGCGGC-3’ |
|  |  |  | 5’-GATCCCGGGCCCGCGGTACCGTCTCCAGGATGGTGGTGGTGGGGTCCC-3’ |
| PRB1 | | NM_005039.4 | 5’-TACCGGACTCAGATCTCGAGCGCCACCATGCTGTTGATTCTGCTGTC-3’ |
|  |  |  | 5’-GATCCCGGGCCCGCGGTACCGTCTGGGGAGGTCTGGAAGGTCTGC-3’ |
| ANXA2 | | NM_001002858.3 | 5’-TACCGGACTCAGATCTCGAGCGCCACCATGGGCCGCCAGCTAGCGGG-3’ |
|  |  |  | 5’-GATCCCGGGCCCGCGGTACCGTGTCATCTCCACCACACAGGTACAG-3’ |
| CAST | | NM_001042440.5 | 5’-TACCGGACTCAGATCTCGAGCGCCACCATGTCCCAGCCCGGCCAGAAG-3’ |
|  |  |  | 5’-GATCCCGGGCCCGCGGTACCGTGTCATCTTTTGGCTTGGAAGTTTC-3’ |
| MARCKS | | NM_002356.7 | 5’-TACCGGACTCAGATCTCGAGCGCCACCATGGGTGCCCAGTTCTCCAAG-3’ |
|  |  |  | 5’-GATCCCGGGCCCGCGGTACCGTCTCTGCCGCCTCCGCTGGGGGGG-3’ |
| EIF4B | | NM_001417.7 | 5’-TACCGGACTCAGATCTCGAGCGCCACCATGGCGGCCTCAGCAAAAAAG-3’ |
|  |  |  | 5’-GATCCCGGGCCCGCGGTACCGTTTCGGCATAATCTTCTCCCTC-3’ |
| XRCC4 | | NM_022406.5 | 5’-GATCCCGGGCCCGCGGTACCGTAATCTCATCAAAGAGGTCTTCTG-3’ |
|  |  |  | 5’-GATCCCGGGCCCGCGGTACCGTAATCTCATCAAAGAGGTCTTCTG-3’ |
| MSI1 | | NM_002442.4 | 5’-TACCGGACTCAGATCTCGAGCGCCACCATGGAGACTGACGCGCCCCAGCCCGG-3’ |
|  |  |  | 5’-GATCCCGGGCCCGCGGTACCGTGTGGTACCCATTGGTGAAGGCTG-3’ |
| TACC3 | | NM_006342.3 | 5’-TACCGGACTCAGATCTCGAGCGCCACCATGAGTCTGCAGGTCTTAAACGAC-3’ |
|  |  |  | 5’-GATCCCGGGCCCGCGGTACCGTGATCTTCTCCATCTTGGAGATGAG-3’ |
| SDCBP | | NM_005625.4 | 5’-TACCGGACTCAGAT​CTCGAGCGCCACCATGGTGAAGCTCGCGAAGGC-3’ |
|  |  |  | 5’-GATCCCGGGCCCGC​GGTACCGTTTCAAACTTCGTCTTCTTTCCTTGTGG-3’ |
| IL22 | | NM_020525.5 | 5’-TACCGGACTCAGATCTCGAGCGCCACCATGGCCGCCCTGCAGAAATC-3’ |
|  |  |  | 5’-GATCCCGGGCCCGCGGTACCGTAATGCAGGCATTTCTCAGAGACATAAAC-3’ |
| MSI2 | | NM_138962.4 | 5’-TACCGGACTCAGATCTCGAGCGCCACCATGGAGGCAAATGGGAGCCAAG-3’ |
|  |  |  | 5’-GATCCCGGGCCCGCGGTACCGTATGGTATCCATTTGTAAAGGCCG-3’ |
| HSPA5 | | NM_005347.5 | 5’-TACCGGACTCAGATCTCGAGCGCCACCATGGAGGCAAATGGGAGCCAAG-3’ |
|  |  |  | 5’-GATCCCGGGCCCGCGGTACCGTATGGTATCCATTTGTAAAGGCCG-3’ |
| PPM1G | | NM_177983.3 | 5’-TACCGGACTCAGATCTCGAGCGCCACCATGGGTGCCTACCTCTCCCAG-3’ |
|  |  |  | 5’-GATCCCGGGCCCGCGGTACCGTGTCTCGCTTGGCCTTCTTCTTCTTGTCG-3’ |
| LIN28A | | NM_024674.6 | 5’-TACCGGACTCAGATCTCGAGCGCCACCATGGGCTCCGTGTCCAACCAG-3’ |
|  |  |  | 5’-GATCCCGGGCCCGCGGTACCGTATTCTGTGCCTCCGGGAGCAG-3’ |
| HSP90AA1 | | NM_005348.4 | 5’-TACCGGACTCAGATCTCGAGCGCCACCATGCCTGAGGAAACCCAGAC-3’ |
|  |  |  | 5’-GATCCCGGGCCCGCGGTACCGTGTCTACTTCTTCCATGCGTGATG-3’ |
| STAT4 | | NM_003151.4 | 5’-TACCGGACTCAGATCTCGAGCGCCACCATGTCTCAGTGGAATCAAGTC-3’ |
|  |  |  | 5’-GATCCCGGGCCCGCGGTACCGTTTCAGCAGAATAAGGAGACTTCATTG-3’ |
| TACC1 | | NM_006283.3 | 5’-TACCGGACTCAGATCTCGAGCGCCACCATGGCGTTCAGCCCGTGGCAG-3’ |
|  |  |  | 5’-GATCCCGGGCCCGCGGTACCGTGTCAGTCTTTCCCAGCTTTGCAATC-3’ |
| BIN2 | | NM_016293.4 | 5’-TACCGGACTCAGATCTCGAGCGCCACCATGGCAGAGGGCAAGGCAGG-3’ |
|  |  |  | 5’-GATCCCGGGCCCGCGGTACCGTGAGTTGTGGATTTTCACTTGTGG-3’ |
